# Green building development utilising modified fired clay bricks and eggshell waste

**DOI:** 10.1038/s41598-025-87435-4

**Published:** 2025-01-27

**Authors:** Wafaa Soliman, Yasser M. Z. Ahmed, Ahmed Ghitas, Abdel‑Hamid El‑Shater, M. Abdelhamid Shahat

**Affiliations:** 1 Geology Department, Faculty of Science, Sohag University, Sohag, Egypt; 2 Refractory and Ceramic Materials Department, Advanced Materials Institute, Central Metallurgical Research and Development Institute, Cairo, Egypt; 3 PV Unit, Solar and Space Research Department, National Research Institute of Astronomy and Geophysics (NRIAG), Helwan, Cairo, Egypt

**Keywords:** Clay–eggshell composite bricks, Eggshell waste recycling, Shrinkage behaviour, Bulk density, Surface porosity, Thermophysical compositions, Environmental sciences, Materials science

## Abstract

The inadequate thermal insulation of the building envelope contributes significantly to the high power consumption of air conditioners in houses. A crucial factor in raising a building’s energy efficiency involves utilizing bricks with high thermal resistance. This issue is accompanied by another critical challenge: recycling and disposing of waste in a way that is both economically and environmentally beneficial, including using it to fuel industrial growth, in order to reduce the harmful effects of waste on the environment as waste generation in our societies grows. To this end, the current study sought to assess whether integrating a specific amount of eggshell waste as CaCO_3_ filler within bricks consistently produces fired clay bricks with desirable thermal insulation capabilities. By systematically investigating the physicochemical and thermal characteristics of bricks doped with varying eggshell content, this work demonstrates how waste materials can be repurposed to produce sustainable construction materials with superior performance. The results highlight significant improvements in thermal conductivity, diffusivity, and effusivity, alongside favorable changes in porosity, bulk density, and mechanical strength. The XRD analysis revealed that once the firing temperature rises, a high insulation feature arises due to siliceous melt formation. EDX analysis gave important insights into the impact of eggshell dopants on the physicochemical parameters of burnt clay bricks. Compared to pristine brick, CEs7% brick constructed with clay and 7 wt% eggshell exhibited a 38.7% loss on dry shrinkage, an enhancement on average pore size of 78.8%, an apparent porosity of 52.7%, a bulk density of 8.3%, and a compressive strength of 57.5%. The reduced shrinkage enhances stability, while increased pore size and porosity improve thermal insulation, making the bricks more durable and energy-efficient. In this regard, the brick containing 10% eggshell that was fired at 1100°C possessed the greatest drop in heat conductivity (i.e., 50%), thermal diffusivity (30%), and thermal effusivity (30%) as compared to the pure one. Given the aforementioned findings, these additions hold the potential to reduce the energy required for both heating and cooling buildings. This brings us to the conclusion that combining eggshell waste to create calcium silicate makes it feasible to be utilized as a thermal insulation material, paving the way for improved construction materials’ performance and sustainability.

## Introduction

Reducing greenhouse gas emissions worldwide through the use of novel green building supplies is an interesting approach, as the building sector is the biggest user of energy, consuming approximately 40% of the world’s energy usage^1^. Therefore, growing energy savings in buildings is an essential objective for sustainable building. Given Egypt’s higher-than-average solar radiation levels^2^, coupled with radiant solar penetration via windows and solar heat gain through the building envelope, plenty of buildings in Egypt are overheated throughout the day. Fully exposed houses require a lot of energy to cool the interior; air conditioners account for the bulk of residential power consumption in this process. For this reason, low thermal conductivity construction materials are essential to minimizing heat flux through the building envelope^3^, especially in hot climates. This coincides with another issue: our societies are experiencing a serious sustainability crisis due to the massive growth in waste production, which produces two billion tonnes of solid trash a year^4^. Considering landfill space is running short and natural ecosystems are being destroyed, there is presently concern regarding how to dispose of these wastes worldwide^5^. To encourage sustainable development, waste should be recycled, reused, and used to create value-added goods.

To address these concerns, it was recently proposed that burnt clay bricks could possess a much-reduced thermal conductivity once correctly treated with waste additives, restricting the amount of heat that passes through building walls. Porous refractory insulation burnt bricks were frequently employed owing to their superior resistance to heat as well as efficacy for delivering insulating properties^6^. The pore-forming elements that form within the burning process are employed to produce insulating clay bricks, since the air trapped in the pores within these porous materials significantly reduces heat transfer^7^. The building insulation qualities of these bricks assist in minimizing undesired heat gain and the amount of energy required by the building’s cooling and heating systems^8^. To attain the goal of thermal insulation bricks, recent research has included a variety of additives within the clay brick matrix^9–13^. According to Sutcu and et al.‘s study^9^, adding waste marble powder to burnt bricks improved the bricks’ porosity, bulk density, and strengths while lowering their thermal conductivity as the amount of waste marble in the brick grew. Date palm seeds were examined by Abu-Jdayil et al.^10^, as a filler in the brick industry. Bricks with high compressive and tensile strength, reduced thermal diffusivity, and thermal conductivity were produced by replacing up to 50% of the natural clay with date palm seeds. Yadav et al.,^11^, explored the potential of recycled paper waste and cotton waste as brick resources; they boosted water absorption and compressive strength, while Ragab et al.,^12^, optimized mechanical, thermal insulation, and physical properties through the integration of pomegranate peel waste (PPW) into clay bricks. Furthermore, Platt et al.,^13^, developed high-insulation prototypes manufactured from bio-based and waste products, including maize pith, repurposed bedding substances, and wheat straw.

**Fig. 1:**
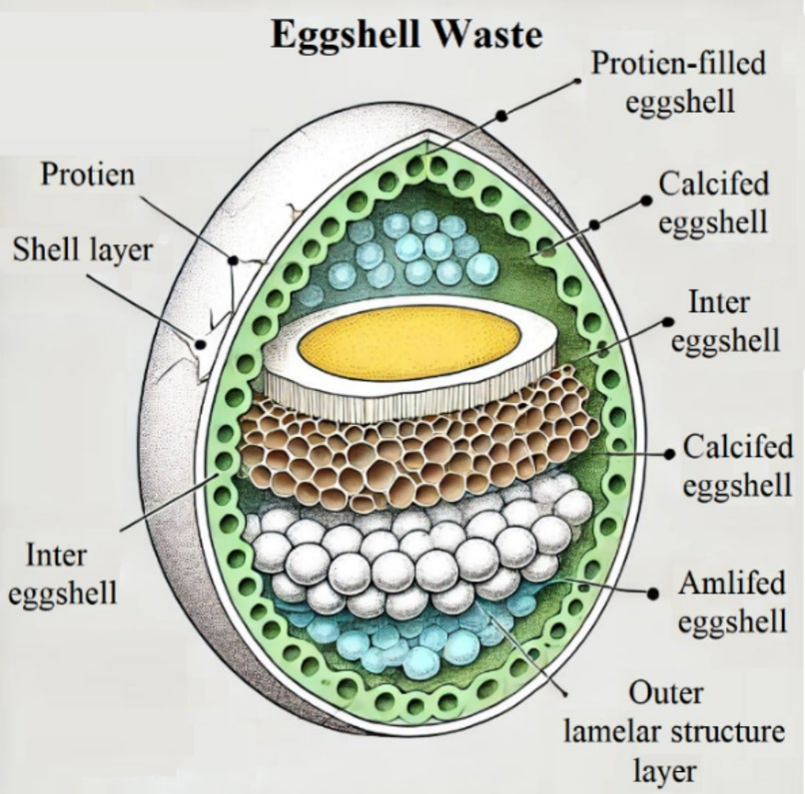
Schematic of eggshell waste composition.

In this context, certain waste products and natural ingredients used for manufacturing bricks possess similar chemical profiles. In light of this, there is an abundance of emphasis on waste recycling to improve the quality of building components by providing an alternate supply of initial components. Based on agriculture, waste and manufacturing byproducts were both categories of trash. Even if industrial processes exhibit superior mechanical performance, recycling agro-based waste offers benefits among them: being inexpensive, readily available, low density, biodegradable, energy-efficient, and ecologically benign. A particular agricultural byproduct item is eggshells, which constitute the hard outer layer of eggs (10% wt egg’s)^14^. Eggshell leftovers are being considered as a prospective raw material replacement for clay brick production^15^. Of the eggshells produced worldwide, over 110 billion end up in landfills^16^. The worldwide egg generation in 2016 was estimated to be 1360 billion eggs, and it is projected to rise by 50% between 2015 and 2035^17^. Egypt produced 510,000 tonnes of eggs in 2016^18^. By 2030, 90 million tonnes of eggs are anticipated to be produced. Such trash has a negative impact on nature since it increases the expense of waste treatment and gives off an offensive odor. The Environmental Protection Agency ranks waste from eggs as the fifteenth most significant contamination concern in the food industry^19^. Considering their numerous advantages, such as their resiliency, affordability, low density, and strong thermal endurance at high temperatures, eggshells are attracting the attention of researchers^20^. For more precise details, most of the content of eggshells is composed of calcium carbonate (i.e., 94% calcite), magnesium carbonate, calcium phosphate, along with organic substances^21^. Two components constitute eggshell trash: a filled-with-protein shell layer and calcified eggshell. Three separate layers form a fresh layer: a spongy middle layer, with a comparable texture to porcelain; a layer within composed of lamellar layers; in addition, an outside layer with a frothy cuticle layer replicated from porcelain^22^. Figure 1 depicts a schematic depiction of the composition of eggshell waste, with clear labelling for each layer and its distinguishing properties. It is often held that eggshells have little commercial value, even though they are rich in minerals, amino acids, and calcium oxide, which make up 94–98% of the eggshell^23^. These enable eggshell recyclables to be utilized as a substitute for natural lime in structures to reduce cement usage while preserving the raw lime. Whereas it is a biomaterial with chemical characteristics akin to those of limestone^24^. Plus, calcium from eggshells is more easily absorbed than calcium found in limestone or coral^25^. In light of these features, integrating eggshell as CaCO_3_ filler to reduce fluxes in major building components serves as one of the most effective techniques for reducing energy consumption and eggshell waste^26^.

Notwithstanding the prospective benefits of using waste materials like eggshell particles as an ecologically friendly resource and reducing environmental waste, a cost-free raw substance, little research was conducted on their influence on the structural and thermal quality of fired clay bricks. Herein, the thermophysical characteristics of eggshell-modified clay bricks were studied, as well as assessments of their microstructure, morphology, density, shrinkage, thermal properties, and porosity. In this regard, our unique clay composites included three new mineral phases (Sanidine, Leucite, and Wollastonite) that are rarely seen. All of these minerals play essential roles in insulation and mechanical strength, with sanidine and leucite-based materials exhibiting excellent mechanical qualities while wollastonite, with low thermal conductivity, is utilized in the creation of thermal insulation materials involving ceramic and foundry lining. This study was conducted with the goal of limiting waste in the environment via using it to produce construction bricks, along with improving the thermal, physical, and structural attributes of these bricks. In the future, these bricks should be suitable for a variety of thermal insulation applications.

## Materials and methods

### Materials

The Kharga Oasis, located between Lat. 25.46–25.78 N and Long. 30.54–30.91 E in the Egyptian Western Desert, provided the clay for this study. This clay, composed of fine crystalline mineral grains, exhibits varied colors, including grey, red, purple, and green. The region near Abu Tartur Mine, formed during the Upper Cretaceous era, is characterized by multicolored claystones interspersed with sandstone pockets, making it a unique and resource-rich area for obtaining clay suitable for construction applications^27,28^. Whereas, Omara et al.^29^ reported that the Quseir Deposit was approximately 30 m thick, with interbedded sandstone, shale, and calcareous sandstone in the upper portion and dark red to reddish-brown claystone in the bottom half. Additionally, the formation was characterized as being nonfossiliferous^29^. On the opposite side, wastes containing broken eggshells are gathered from nearby sources. Eggshells were gathered, cleaned, and then dried in the sun for two days before being ground into a powder and placed in boiling water for five to ten minutes.

### Clay bricks design and shaping behaviour

**Fig. 2:**
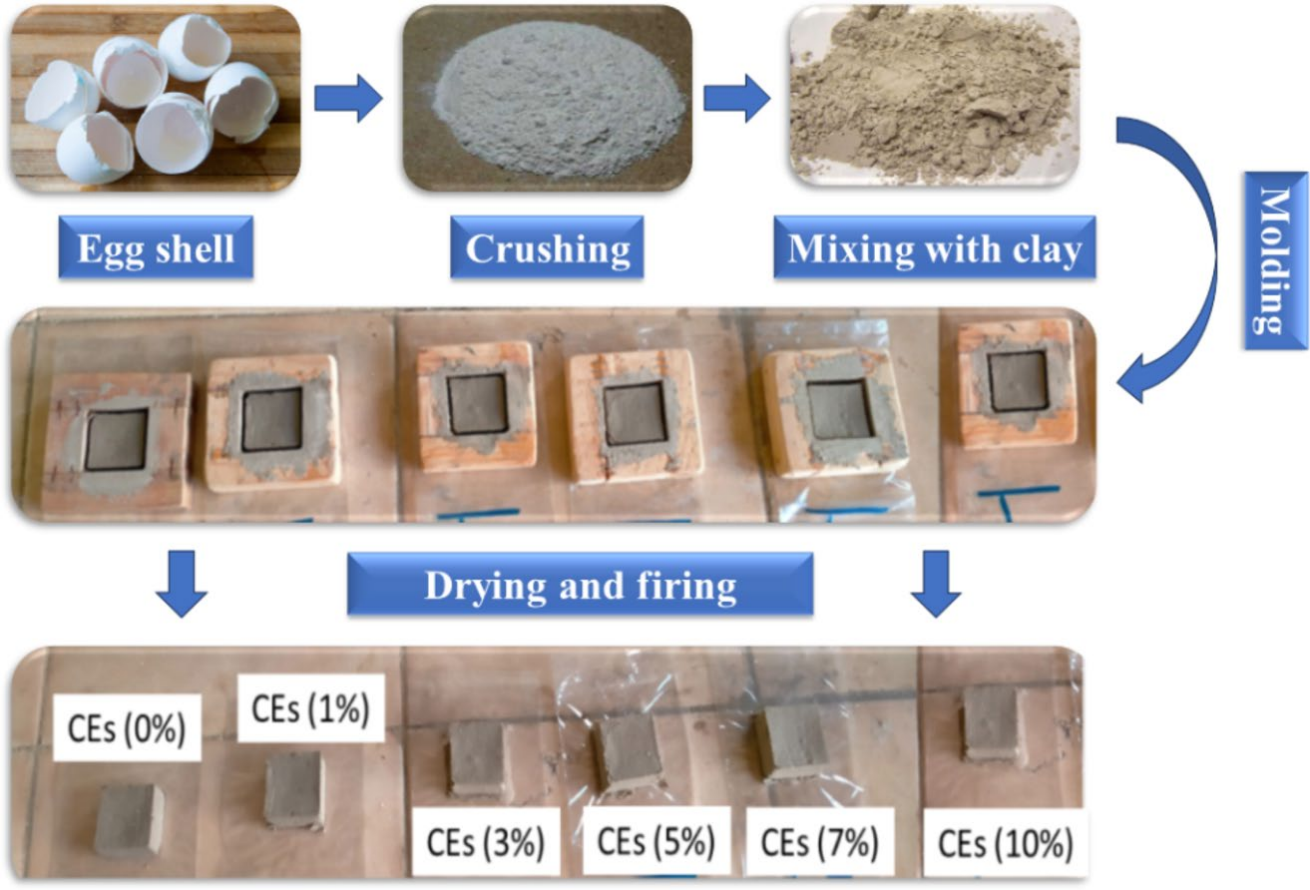
A series of steps for the designing clay bricks.

In the brick-making process, eggshell additives were incorporated with clay soil and utilized as depicted in Fig. 2. Manual molding of test pieces of clay was done in the lab using a wooden mold. The newly constructed raw bricks (4 cm x 4 cm x 2 cm) were naturally dried at room temperature for 3 days before being fired. This was done in order to ensure there was no moisture present before firing and to protect bricks from bending and splitting while fired at a very high temperature. The bricks were fired for 4 h at a temperature of 1100°C in a muffle furnace that provided an oxidizing environment. The above-raised vitrification degree was rendered possible by the presence of alkali oxides in the fired bricks, which made them harder and less prone to weathering and differential moisture absorption. High firing temperatures also cause a number of chemical reactions, such as the disintegration of quartz and the enhancement of the bricks’ degree of verification, which endows them with excellent mechanical and thermal qualities. In this regard, the bulk mineralogy of the sample includes phyllosilicates (30%) and k-feldspar (70%), and the mineralogy of the clay is smectite/illite mixed layers (93%) and kaolinite (7%). Herein, clay behaviour during forming was assessed using plasticity measures. The sample was constructed of 40 g of tempering water and 50 g of clay. Following this, in various quantities, the eggshell powder additions were applied to the specimens. The terms used include CEs0%, which refers to unprocessed brick, and CEs1%, CEs3%, CEs5%, CEs7%, and CEs10%, which refer to treated bricks. Each of them was employed to donate various percentages of eggshell blended with the clay matrix. In this regard, every sample was shaped, dried, and fired without any cracks. They all functioned perfectly. With the exception of bricks coated with more than 10% eggshell, which function well while drying but disintegrate after firing. The description assignments for clay-based bricks based on various eggshell constituent amounts are listed in Table 1.

**Table 1:** Description assignments for clay-based bricks modified with different amounts of eggshells materials.

Sample Code	Clay amount (g)	Eggshells percentage	Curing (Firing) temperature	Curing (Firing) time	Description
CEs0%	50.0	0%	1100^°^C	4 h	Clay–Eggshells (0%)
CEs1%	49.5	1%	1100^°^C	4 h	Clay–Eggshells (1%)
CEs3%	48.5	3%	1100^°^C	4 h	Clay–Eggshells (3%)
CEs5%	47.5	5%	1100^°^C	4 h	Clay–Eggshells (5%)
CEs7%	46.5	7%	1100^°^C	4 h	Clay–Eggshells (7%)
CEs10%	45.0	10%	1100°C	4 h	Clay–Eggshells (10%)

### Bricks characterization

By employing X-ray Diffractometry (XRD) with a Cu target (Model D8, Bruker), patterns from 4 to 70° 2θ were detected, providing insight into the phase development and mineral structure of the pure and processed clay compounds. Clay grain changes in structure as a consequence of chemical treatment were investigated using Fourier transform infrared (FTIR) spectroscopy (Jasco Model 4100, Japan). At ambient temperature, data with a 4 cm^–1^ resolution in a 4000–400 cm^–1^ region are produced by the IR spectrum. Assessing the clay brick’s fluctuation in sizes during drying and firing at room temperature allowed for the evaluation of the shrinkage behaviour resulting from these processes. The surface morphology of the hybrid bricks was further examined using field emission scanning electron microscopy (FESEM, QUANTA 200 FEG from FEI, Japan) equipped with an Energy-dispersive X-ray (EDX) spectrometer to identify the surface chemical composition of the components. Moreover, physical measurements of apparent porosity and bulk density were made using the Archimedes method. The compressive strength was evaluated utilizing EN 772-1:2011, with each of the samples gradually increasing in strength through placing a load centered on its top surface until failure. The compressive strength of each material was subsequently assessed and reported in MPa. The thermogravimetric analyzer (TGA) 1600 SETARAM LABSYS Evo was utilised for a thermal stability examination, which was carried out within the temperature range of 23 to 1000 °C. With an isothermal precision of ± 1 °C, the cooling and heating rates in the air surrounds were both 10 °C/min. The thermal parameters of the fired clay bricks, including thermal conductivity, thermal diffusivity, specific heat capacity, and thermal effusivity, were measured using standard techniques to evaluate the insulation performance. The measurement procedures were evaluated using a Hot Disc Thermal Constants Analyzer (Hot Disc TPS 2500 S, Göteborg, Sweden). This instrument employs the transient plane source (TPS) technique, which is well-known for its accuracy and convenience in investigating thermal transport properties. Samples were prepared in standard sizes and tested at room temperature. The samples were placed in contact with a sensor, and thermal conductivity was calculated using the heat flow response over time. On the other hand, a heat pulse was applied to one side of the sample, and the temperature rise on the opposite side was measured to determine diffusivity. In addition, specific heat capacity was assessed by heating the samples and measuring the heat required to raise their temperature to determine the specific heat. Thermal effusivity was calculated using the material’s thermal conductivity, density, and specific heat capacity.

## Results and discussion

### XRD and FTIR spectra

**Fig. 3:**
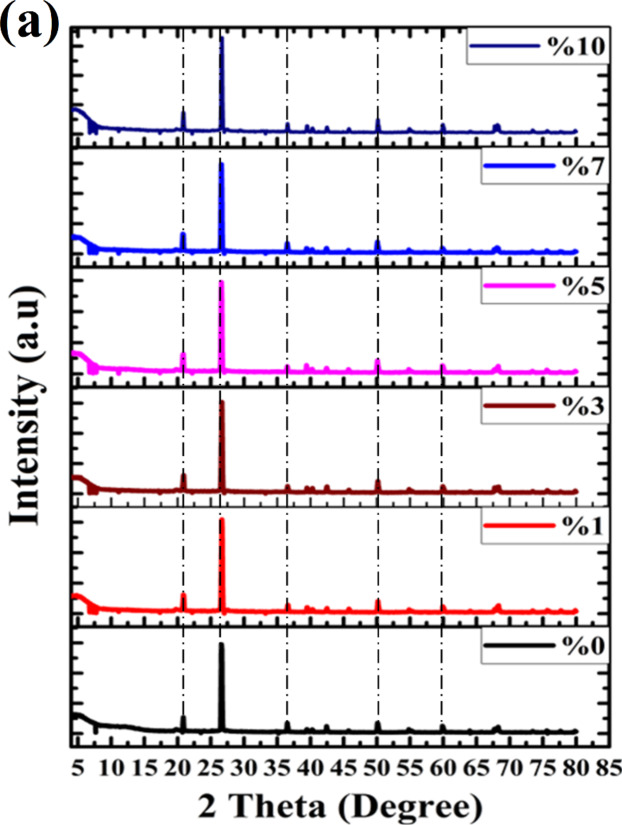

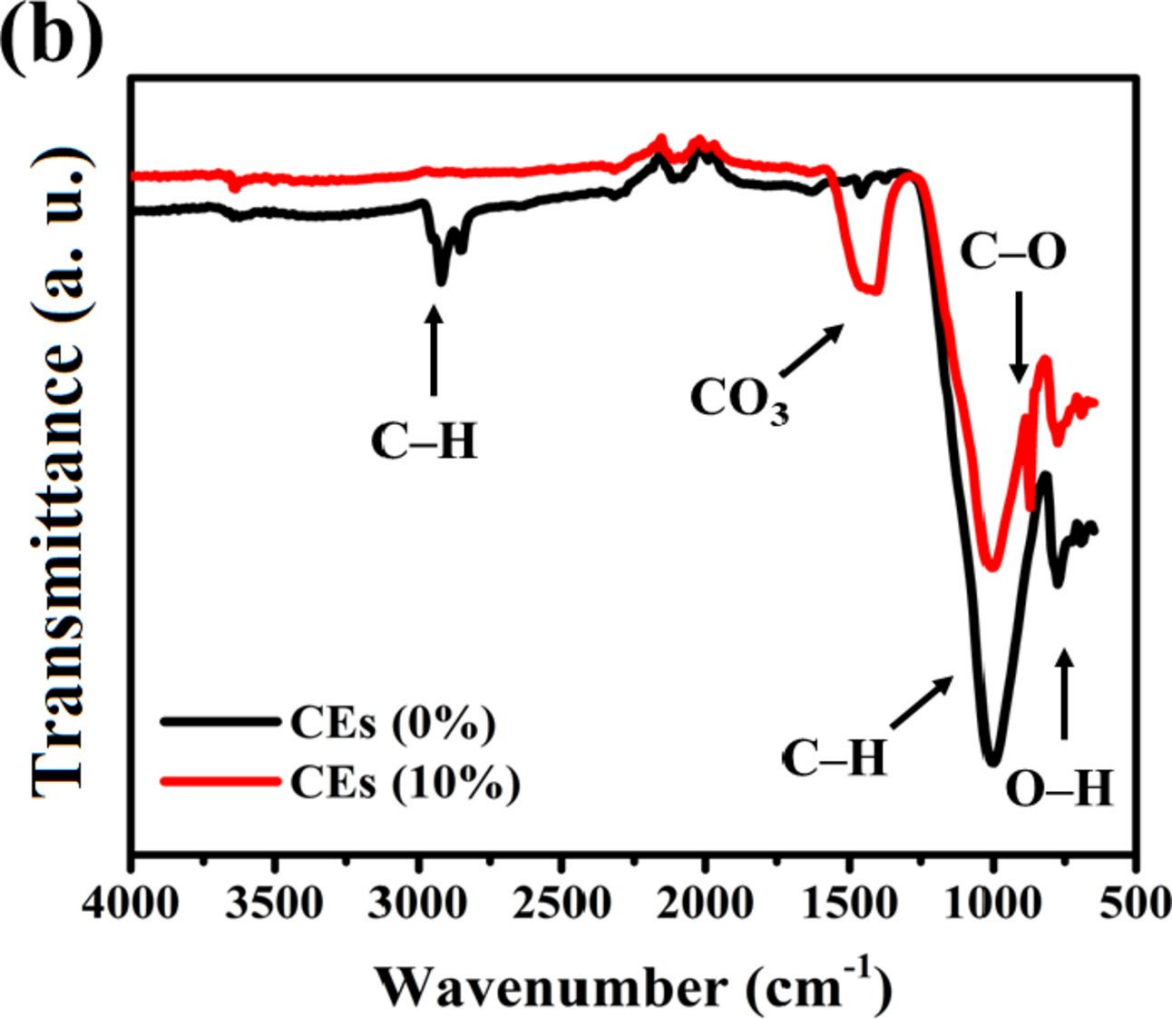
(**a**) X-ray diffraction patterns, (**b**) FTIR spectra of untreated and treated clay using various ratios of eggshell.

Figure 3a and b depict the microstructure changes, including X-ray diffraction patterns and FTIR spectra of burnt clay composites loaded with various amounts of eggshell dopants. Phyllosilicates are the major constituents of the raw materials used for producing clay bricks, as seen in Fig. 3a. Feldspar can be observed at 2θ = 21.8^°^^30,31^, eggshell Calcite (Joint Committee on Powder Diffraction Standards (JCPDS) card No: 05–0586) at 2θ = 28^°^, Kaolinite (JCPDS card No: 00–005–0143) at 2θ = 12.2^°^, and combined illite/smectite at 2θ = 8.4^°^. Upon burning at 1100 °C with and without additives, the predominant mineral phase present in these bricks is Quartz (JCPDS card No: 00–046–1045). The Mullite A_l6_Si_2_O_13_ was formed also at 2θ = 17^°^, 26^°^, 33^°^, 37^°^, and 40^°^ (JCPDS card No: 15–776). In these bricks, 2θ = 28^°^, 36^°^, 39^°^, 40^°^, 46^°^, and 50^°^ crystallized, and the peaks of clay minerals, especially kaolinite disappeared^32^. The carbonate begins to disintegrate and release CO_2_ among 600^°^C and 850^°^C. The interaction of silicates with calcium oxide results in the creation of diverse mineral phases. Leucite (K_2_O.Al_2_O_3_.4SiO_2_) (JCPDS card No: 38–1423) is formed when potassium feldspar (K_20_A1_2_O_3_SiO_2_) melts. This high-temperature phase has 2θ = 7^°^, 13^°^, and 17^°^ and is temperature-resistant, improving the mechanical properties of bricks and possessing a high siliceous melt^33^. The level of amorphous component grows with increasing fire temperature, suggesting enhanced vitrification of the bricks, which leads to better mechanical and advantageous thermal properties^34^. Still, some leucite (3KAlSiO_8_) turns into sanidine (JCPDS card No: 19–1227), 7.6^°^, 11^°^, 17^°^, 21^°^, and 25^°^ as the temperature rises^35^. Ceramics that are based on sanidine have outstanding mechanical qualities^36^. When phyllosilicates decompose and combine with carbonate to form new Ca-silicate minerals, Wollastonite (CaSiO_3_) is produced (JCPDS card No: 003–0559). Thermal insulator and industrial lining ceramic materials are made using low thermal conductivity ceramic substances with 2θ = 23^°^, 26^°^, 36^°^, and 43^°^^37,38^.

Nevertheless, the FTIR spectra presented in Fig. 3b indicate that the absorption band near 781.9 cm^–1^ corresponds to the O–H stretching vibration of the structural water^39,40^; the band around 875 cm^–1^ is associated with the vibration bands of the C–O bonds of calcite^41^, while a band at 998 cm^–1^ is related to the C–H bending^42^. In carbonate, an asymmetric stretch band of CO_3_ was discovered at 1400 cm^–1^, corresponding to the appearance of the C–O group’s stretch vibrations at 1006 cm^–1^^43^. The C–H stretching band is located at 2853 cm^–1^ and 2922 cm^–1^^44^. Whereas the incorporation of eggshell carbonates into the brick’s structure is what causes the appearance of carbonate bands. Experimental data indicates the presence of a thin layer of hydroxides (O–H) on the surface of calcium carbonate. As a result of a contact involving the active hydrogen of a C–H bond and the hydroxyl ions on the surface of CaCO_3_, C–O bonds are formed by the molecules^45–47^.

### Shrinkage behaviour

One very essential attribute of clays required for producing bricks is their capacity to shrink during the drying and burning processes. Where clay shrinks when it dries varies on a number of variables, including the amount of extremely minute colloidal particles and the existence of certain minerals. Murray^48^, states that substantial shrinkage, cracking, and drying delays occur when montmorillonite is found in quantities of 15–25% or more. Shrinkage results from the removal of water from a ceramic body during drying. To avoid internal faults, the rate at which water gets eliminated during the drying process must be carefully regulated. The drying shrinkage for bricks of sufficient quality should be about 10%. With respect to the amount of volatile compounds present, it determines the sorts of crystalline phases that compose the brick, as well as how effectively the clay minerals dehydrate, causing a body to shrink or expand when burned. Murray^48^, also asserts that fire shrinkage can additionally be represented in terms of volume or linear dimensions. Moreover, the composition, shape, and size of the clay mineral particles influence shrinkage. The clay bricks shrink as water in the clay matrix evaporates during the burning cycle. Whenever the water surrounding the particles evaporates, they shrink and grow closer together^49^. Monitoring the level of burning shrinkage allows one to ensure the quality of burnt clay bricks. Normally, a good brick shrinks by less than 8%^50^. The final strength of the clay brick is affected by temperature. Strength improves when additional elements melt at greater degrees. The specimens handled with eggshell powder demonstrate adequate drying and firing shrinkage, with no fractures resulting from shrinking as listed in Table 2. In the first procedure, the orientation of the chipping in damp bricks, whose are made by tossing clay into molds, becomes more random during the drying stage, resulting in more isotropic behaviour. As soon as the created brick dried, its volume decreased (by at least 9.2% at CEs7%), and a portion of early strength evolved. As a consequence, the interior of the brick shrinks more slowly than the exterior. Furthermore, shrinkage fractures may form if water evaporates too quickly, lowering strength considerably^51^. Afterwards, during the fire phase of this work, the shrinkage achieved was 2.9% of the pristine mixture, which eventually dropped to an ideal value of 2.8% with the inclusion of CEs7% hybrid brick. This reduction in firing shrinkage enhances the brick’s insulating properties by improving dimensional stability, controlling porosity for better thermal insulation, and maintaining structural integrity. This balance ensures efficient insulation without compromising strength. These enhancements might be attributed to the hydrophobicity of CaCO_3_ and calcite minerals and the filler-matrix adhesion/interlocking generated through their wrinkled appear, which has superior two-dimensional structure^52,53^.

**Table 2:** Drying and firing shrinkage of prepared bricks at different ratio of eggshell.

Sample	Drying shrinkage (%)	Firing shrinkage (%)
CEs0%	15	2.9
CEs1%	9.2	0.9
CEs3%	10	1.9
CEs5%	12.5	1.96
CEs7%	9.2	2.8
CEs10%	12.5	1.96
CEs15%	1.6	Damaged during firing
CEs20%	10	Damaged during firing

### Morphology

**Fig. 4:**
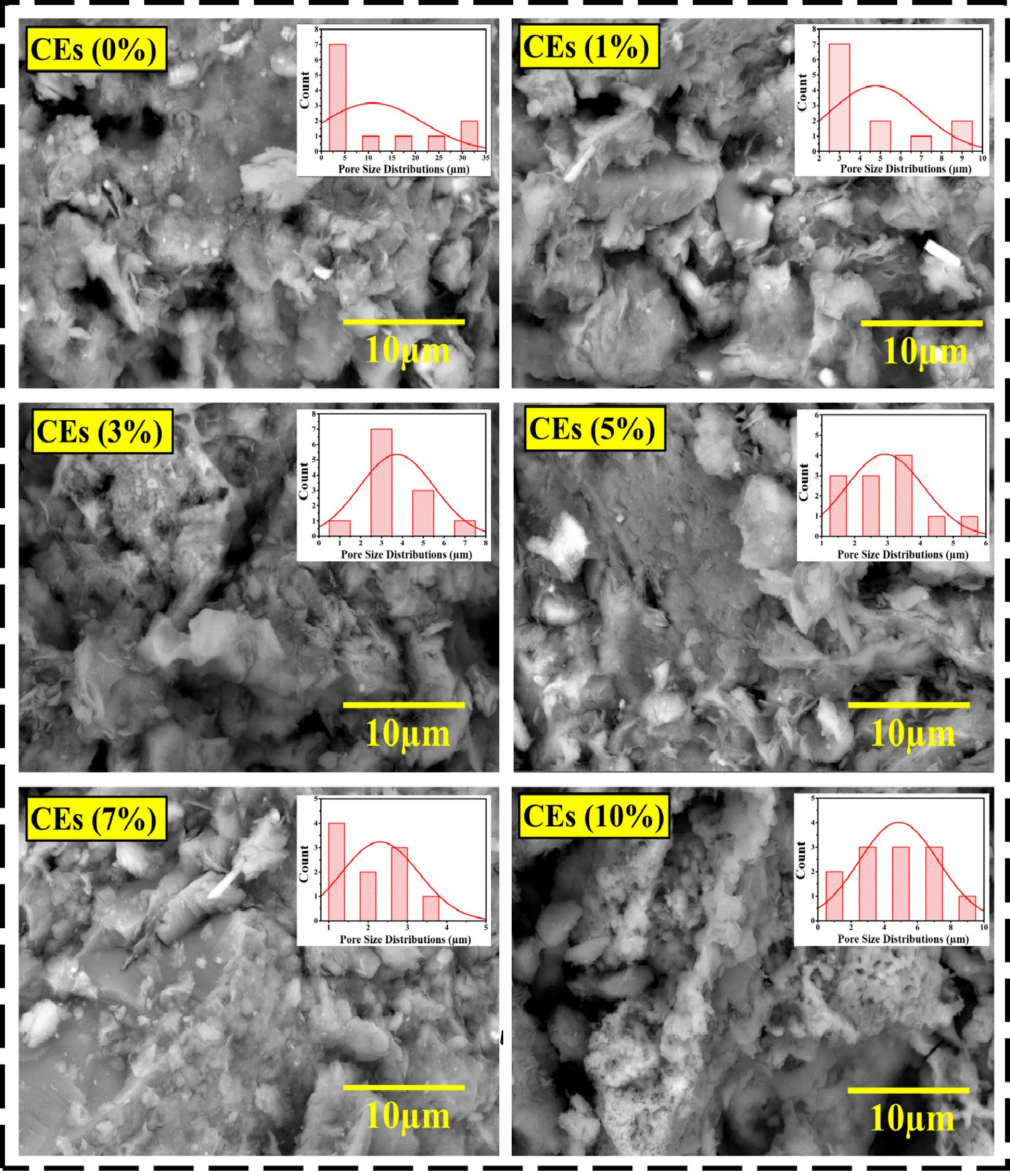
SEM micrographs and statistical histograms of pore size distribution of pristine and modified composite clay with varying eggshells waste ratios.

Figure 4 shows SEM micrographs and pore size distributions of unprocessed and processed clay compounds with different eggshell waste inputs. The porous topology of the clay composites is observable, contingent on the degree of doping. Whereas CEs0% depicts the surface properties of the pure, fine-pored clay. Moreover, comparable structures may be observed in wastes and clay that were introduced in different levels of CEs (1, 3, 5, 7 and 10%). It was discovered that the active hydrogen bonds C–H generated and the abundant basic hydroxyl groups of eggshell material are distributed fairly throughout the clay structure, resulting in strong filler adhesion^54^. Further, at growing eggshell waste levels, the compounds’ structure expanded to disclose an amorphous structure with larger micropores, numerous linkages, and increased internal energy^55,56^. In that time, a number of crumpled and wrinkly structures appeared in the clay blends. The porous nature of clays is essential for limiting individual nanosheet aggregation and unwanted restacking^57^. In this regard, Table 3 tabulates the statistical estimates of the pore size distribution and average pore sizes of both untreated as well as treated mixed materials. The average pore size of pure clay CEs0% was 10.92 μm, which dropped to 4.76 μm after adding (1%) CEs wastes into the clay matrix. This pattern persisted as the dopant ratios increased (i.e., CEs3% = 3.73 μm, CEs5% = 2.93 μm), with the CEs7% brick yielding the best result of 2.31 μm. Nevertheless, adding more eggshells to the 10% blend of CEs possessed the opposite effect, increasing the average pore size to 4.87 μm. The formation of entirely novel intermolecular covalent bonds, hydroxyl ions on the surface of CaCO_3_, hydrogen bonds with clay–H_2_O, –OH groups, and C atoms forming C–O bonds were potential causes for these results^45,58^. These interconnections allow the eggshell waste within clay mixtures to be distributed uniformly and have an impact on the pace at which hydration crystals develop^59,60^. The miscibility of the components improved surface roughness (Talysurf 50 profilometer, Taylor Hobson Precision). The average surface roughness parameter (Ra) rose from 12.8 μm in CEs0% to 19.3 μm in CEs10%, as well as the thermal properties of these hybrids^61,62^.

### Energy Dispersive X-ray (EDX) analysis

**Fig. 5:**
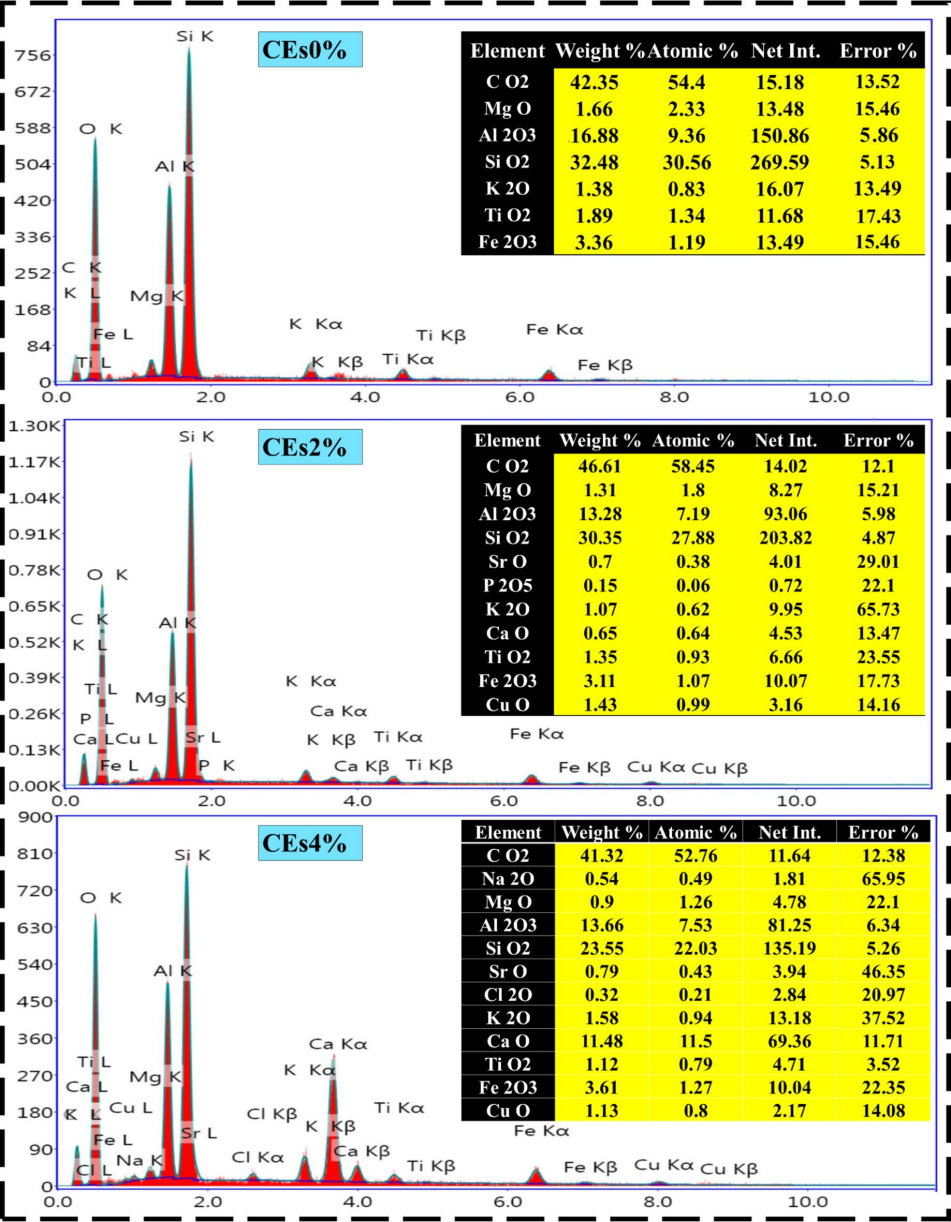

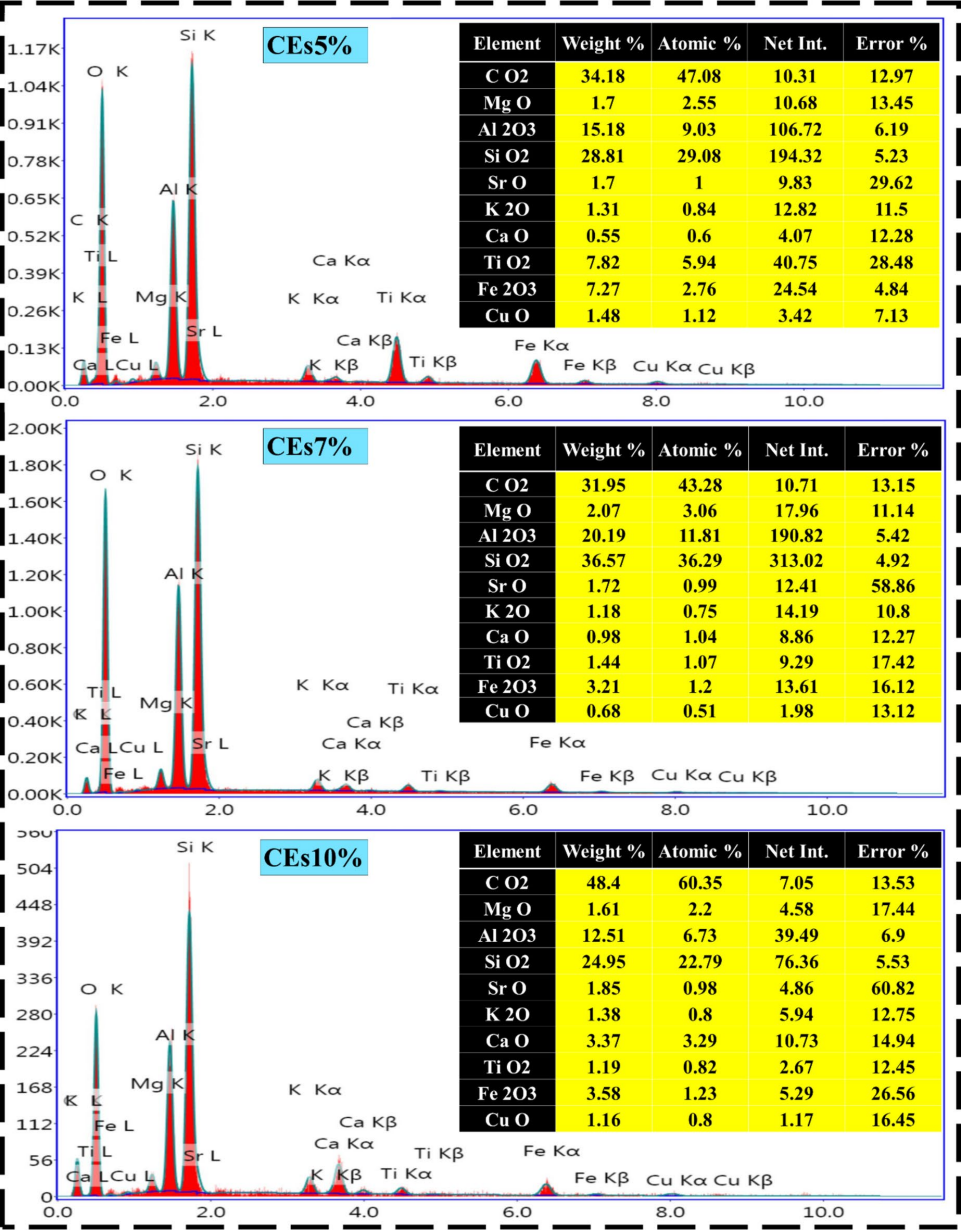
EDX observations of pristine and modified composite clay with varying eggshells waste ratios.

An essential method to improve the elemental composition of bricks and hence their chemical, physical, and mechanical qualities over regular clay bricks is to include eggshell dopants into the burnt clay brick system^63^. In this section the influence of varied levels of eggshell dopants—which are mostly made of CaCO_3_—on the thermophysical performance of clay bricks was examined using EDX analysis, as displayed in Fig. 5. In varied ratios, elemental maps of pristine clay brick revealed the distribution of key elements including, CO_2_, MgO, Al_2_O_3_, SiO_2_, K_2_O, TiO_2_, and Fe_2_O_3_^64^. The treated clay bricks remained this frame structure of elemental compositions alongside adding other minerals including, SrO, P_2_O_5_, CaO, CuO, and Na_2_O in accordance with the eggshell ratio^65^. In this regard, the 7% eggshell ingredient in the CEs7% composite was sufficient for optimizing the weights of several essential minerals, such as SiO_2_ (36.57 wt%) and Al_2_O_3_ (20.19 wt%), while reducing CO_2_ (31.95 wt%), K_2_O (1.18 wt%), and CuO (0.68 wt%) compounds. Besides that, elemental composition EDX spectra revealed that the calcium (Ca) concentration rose in accordance with the level of eggshell doping (i.e., from 0 wt% in the CEs0% hybrid up to 11.48 wt% in the CEs3% compound)^66^. Calcium was distributed uniformly throughout the clay matrix at lower dopant levels, whereas calcium segregation zones, suggesting potential phase separation, were seen at higher dopant levels. Chemically, the addition of Ca from eggshells resulted in the creation of new Ca-rich phases, which may include calcium silicates and increase the strength of the brick^66^. While possible modifications to the brick’s microstructure and phase composition were suggested by the elemental redistribution and the interaction between Ca and other elements (Si, Al)^65^. Integrating SEM pictures with EDX mapping revealed variations in porosity based on the dopant levels, particularly those driven by the release of CO_2_ during the decomposition of CaCO_3_^67,68^. Excessive doping (≥ 7%) caused calcium segregation and increased porosity, possibly weakening the brick. By leveraging EDX analysis, this study provided insights into how varying levels of eggshell dopants affect the chemical and physical properties of fired clay bricks at 1100 °C.

### Porosity, bulk density and compressive strength analysis

Given the significance of porosity, which is consistent with morphology and EDX observations, the apparent porosity and density parameters of the synthesised compounds were determined using the Archimedes approach. Given that they are among the most significant variables affecting thermal insulation^69^. Based on the eggshell content and fire temperature, the measured values of the brick’s porosity and bulk density were revealed as tabulated in Table 3. Whereas with the eggshell percentage rising to 7%, the bricks’ porosity nature declined from 28.07 to 13.28%. Moreover, the tendency was reversed by increasing the amount of dopant waste, with apparent porosity at the CEs10% specimen rising to 38.5%. Higher porosity and lower density of the samples were a consequence of the increased quantity of CO_2_ emitted due to the increased eggshell composition of the bricks^20,70^. Every aspect considered, The carbonate content of the brick material influences the creation of pores during burning; at high temperatures, calcite breaks down into lime (CaO) and CO_2_ gas, and the subsequent evolution of CO_2_ gas decreases porosity^71–75^.

Light-density bricks provide more opportunities for thermal insulation than normal-weight bricks^76^. Hence, for all designed bricks, raising eggshell content resulted in a decrease in brick density, highlighted in Table 3. The bulk density of the pristine material of CEs0% was 1.81 g/cm^3^, and declined to 1.56 g/cm^3^ with an eggshell level of up to 10% in CEs10% specimens. These results are supported by the differ-dense microstructure presented in the SEM images^77^. Further, finer particle development drives densification by increasing the rate of atomic diffusion, resulting in denser microstructures and driving fluctuations in the density trend^78^. In the meantime, given the chemicals’ tendency to decompose during their combustion cycle, the density of bricks carrying additives falls^79,80^. More specifically, the bloating impact and calcite degradation during elevated firing temperatures led to a rise in porosity, which in turn affected the decreasing bulk density of bricks. Carbonates decompose at high temperatures, producing CO_2_. Given the higher vitrification and densification at 1100^°^C brought about by the release of CO_2_, the brick develops pores and capillaries, which lowers density and increases thermal insulation and is thought to be an energy-saving technique^7,81^. Additionally, while eggshell ashes possess a lower specific gravity than clay, replacing the clay with them at a temperature of 1100^°^C drops the density. Lightweight bricks, resistance to moisture, decreased shrinkage and cracking, decreased thermal expansion, cost savings, energy-efficient burning, sustainability, sound absorption, and thermal insulation in structures are some of the advantages^74^. On the other hand, increasing the number of air pockets within the brick improves structural insulation by controlling temperature and consuming less energy. Prior to that, research utilising a variety of dopants, including TiO_2_ NPs and fly ash, demonstrated a comparable pattern of bulk density behaviour^82,83^.

In this vein, a rise in eggshell causes a loss in compressive strength as it causes calcite to decompose and bloat, thereby increasing the number of pores and weakening the bond within the brick structure^84^. Nonetheless, when a thick structure forms, raising the firing temperature increases the compressive strength. Similar results were observed when carbonate was added to clay bricks^85^. As the quantity of eggshell grows, the resulting ceramic bricks’ compressive strengths range from 1.177 MPa to 0.05 MPa. The unprocessed sample that was sintered had the highest compressive strengths, whereas the 10% eggshell bricks exhibited the lowest compressive values. Clay bricks having a compressive strength of at least 20 MPa are classified as first-class clay bricks, meaning they can withstand severe weathering, as per the ESS-4763 (2006) and Standard Specification for Building Brick. Compressive strength of less than 10 MPa is classified as third-class brick, while that of the second class is defined as those that can withstand typical weathering. This suggests that the third category is where those who received eggshell dopants fall in the fired brick structure used in this research as listed in Table 3ref^86^.

**Table 3:** Physical observations on the porosity, bulk density, compressive strength, and average pore size of both pure and modified composite clay containing various eggshell waste ratios.

Sample No.	Apparent porosity (%)	Bulk density (g/cm^3^)	Compressive strength (MPa)	Average pore size (µm)
CEs0%	28.07	1.81	1.177	10.92
CEs1%	16.72	1.63	0.69	4.76
CEs3%	11.24	1.84	1.1287	3.73
CEs5%	32.11	1.71	1.113	2.93
CEs7%	13.28	1.66	0.5	2.31
CEs10%	38.50	1.56	0.05	4.87

### Thermogravimetric analysis (TGA)

**Fig. 6:**
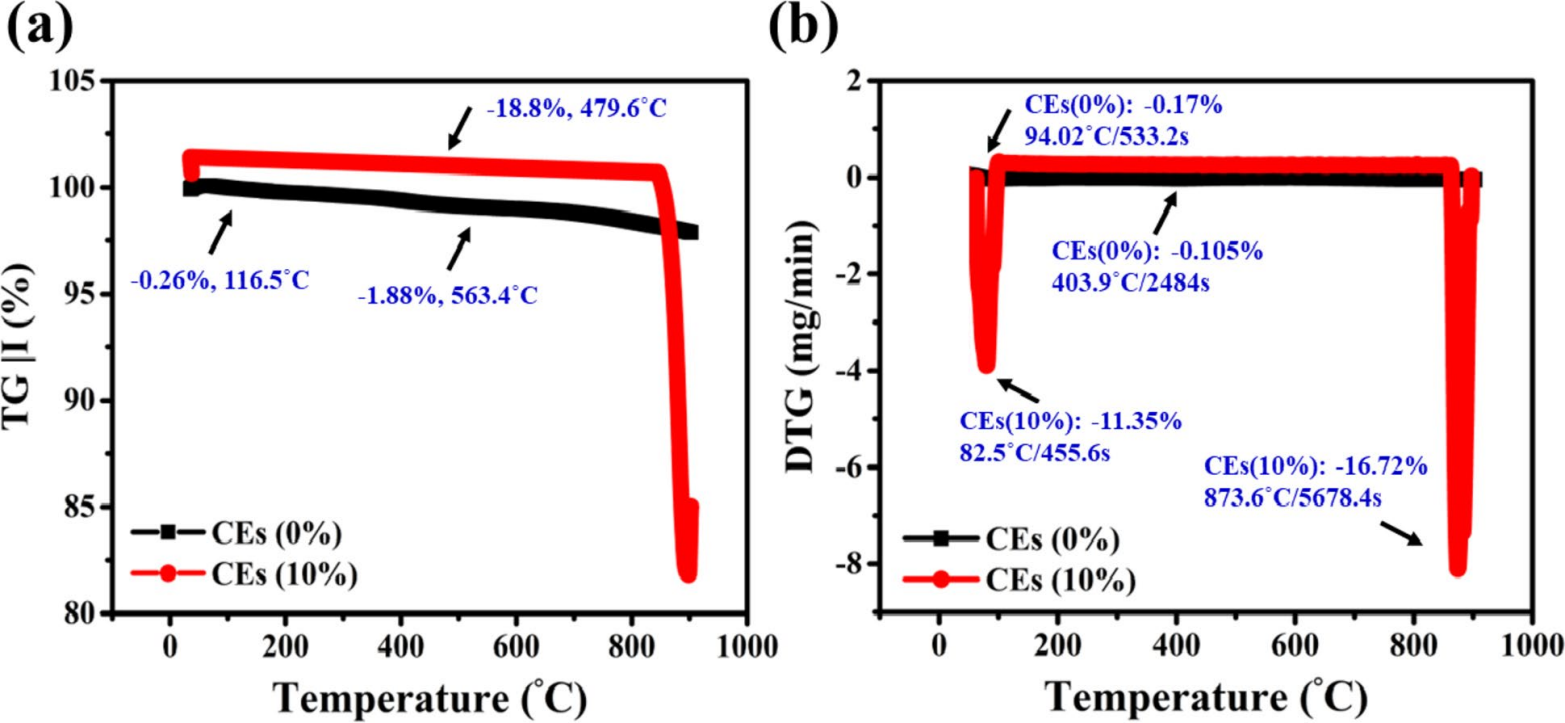
(**a**) TGA, (**b**) DTG curves of pristine and treated clay using various ratios of eggshell waste.

Based on the level of CEs utilised as a dopant substance component, TGA and DTG techniques were utilised to assess the thermal stability of the processed and unprocessed clay compounds, as displayed in Fig. 6. The dehydrated state of elements in clay was primarily induced by molecules of water that were incorporated into the clay structures, as Fig. 6a and b exhibit. This consists of actively received water along with trapped water that acts with interface cations in the 25–900 ˚C region^87^. As a consequence, the physical weight of the modified clay components (CEs10%) decreased by − 18.8% at 479.6 ˚C, whereas the pure (CEs0%) declined by − 0.26% and − 1.800% at 116.5 ˚C and 563.4 ˚C, respectively. A hydroxide ion O–H on the surface of CaCO_3_^39^, and the active hydrogen C–O bonds of calcite^45^, chemically interacted as a result of the shift brought about by the presence of CaCO_3_ extracted from eggshells within clay layers. Other functional groups, such as C–H^42^, were also added. The data collected is practically supported by in-situ XRD patterns^88^. Additionally, the CEs10% instance reveals a broad and high intensity of the peak (i.e., − 11.35% weight loss) at 82.5˚C/455.6s, whereas the pure material recorded a tiny intensity of the DTG peak at that same temperature. Material weight masses dropped by − 0.17% at 94.02˚C/533.2s in the CEs0%. The continuing existence of oxygen-containing chemicals generated from eggshell wastes, among them carboxylates and hydroxyl, whose pyrolyzed and released CO, CO_2_, and H_2_O vapour, is accountable for the expansion^89^. Similarly, between 300 and 880 ˚C, the primary cause of weight losses within both injected and raw mixtures is kaolinite dehydroxylation^90^. Consequently, pure clay lost mass by − 0.105% at 403.9˚C/2484s. Possible extra clay minerals that dehydroxylate in this temperature range include smectite and illite/mica, which might very slightly affect the weight loss. The DTG profile of the CEs10% hybrid revealed a notable peak at 873.6˚C/5678.4s, with − 16.72% weight losses, indicating that the most robust groups of nanofiller sheets—such as carbonyl and quinone—were disintegrating. This displayed peak is attributed to the breakdown of calcite and the dehydroxylation of illite^91^. Results similar to mullite or γ-alumina nucleation acceleration were obtained in this experiment using a ramp rate of 10 ˚C/min. The remaining mass weights of both the treated and pristine clay elements throughout the temperature range of 25 to 870˚C. This implies that the weight loss of eggshells-containing clay bricks is somewhat higher. Consequently, the excellent thermal stability and resistance to thermal shock of these burnt clay bricks result in exceptional building durability.

### Thermophysical characteristics

**Fig. 7:**
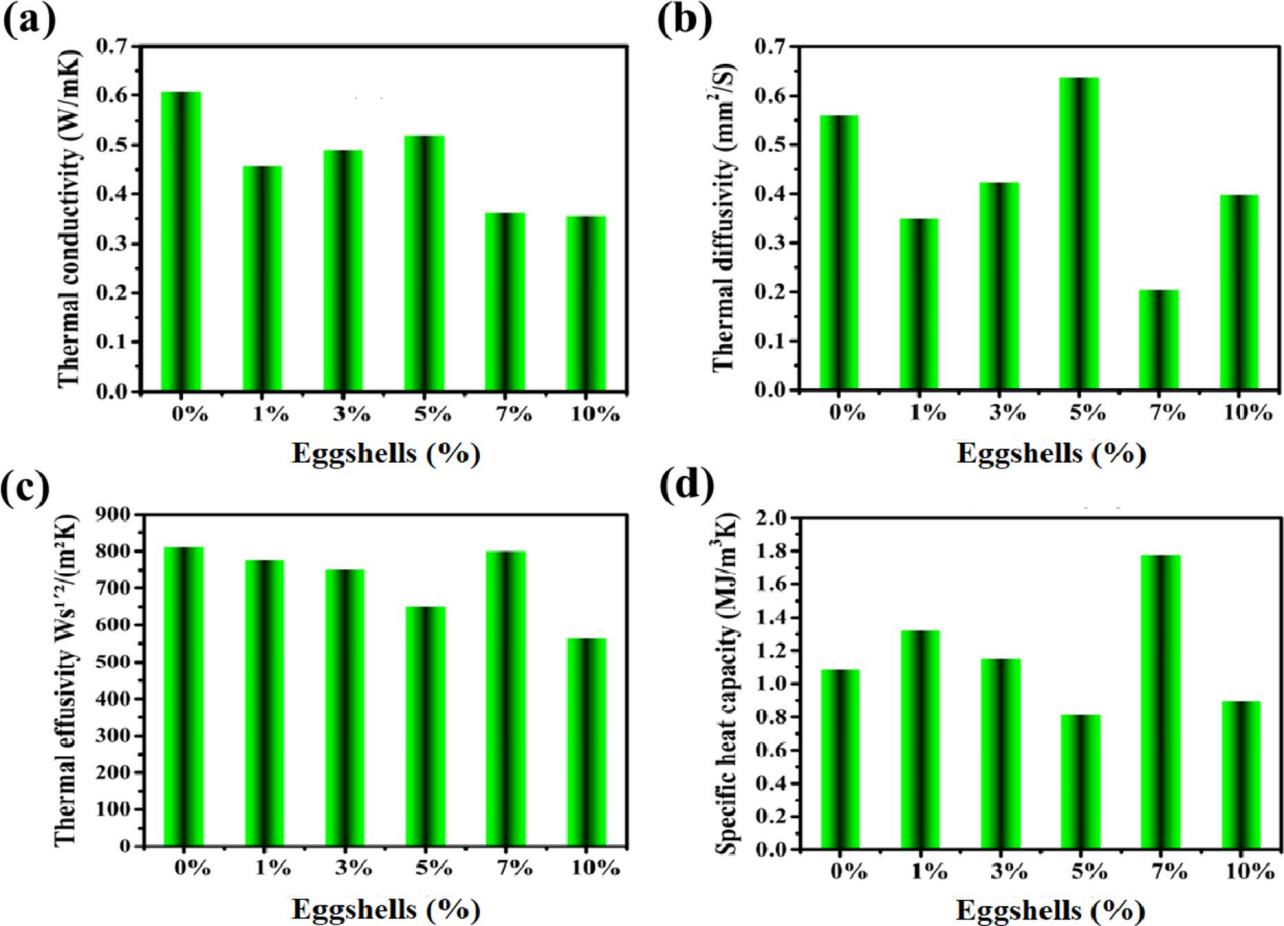
Thermal and physical characteristics of the designed bricks.

The level of eggshell dopants employed possesses a major impact on the thermal properties of clay bricks. Thermal conductivity, thermal diffusion, thermal effusivity, and specific heat capacity are among the thermophysical activity improvements that are covered in Fig. 7a, b and c, and 7d.

#### Thermal conductivity

With varying ratios of eggshell (1, 3, 5, 7, 10%), the thermal conductivity of both treated and untreated bricks is displayed in Fig. 7a. Thermal conductivity clearly reduces from 0.6081 W/mK to 0.3562 W/mK, when eggshell levels rise from 0 to 10%, respectively. This falls within the UNE-EN 1745 standard’s specified range (< 0.430 W/mK)^92^. In view of the calcite degradation in the clay matrix related to pore development, raising the additives concentration lowers the heat conductivity. This brings us to the conclusion that combining eggshell waste to create calcium silicate makes it easy to utilize as a thermal insulation material^93^. The creation of micropores in the brick body during the sintering process, caused by the burning of organic components, produces bricks with significantly better thermal insulation. Plus, the thermal conductivity is positively impacted by the firing temperature. Raising the firing temperature promotes densification and the formation of the liquid phase in the brick substance, thereby lowering heat conductivity. The brick with 10% eggshell that was burnt at 1100^°^C showed the largest 50% decrease in heat conductivity. Given its ability to lower the energy required for both heating and cooling buildings, low thermal conductivity is essential^94^.

#### Thermal diffusivity, effusivity and specific heat capacity

The inclusion of multiple eggshell fillers in a clay composition reduced both thermal diffusivity and thermal effusivity characteristics. Whereas in Fig. 7b, the thermal diffusivity of planned bricks fell from 0.5610 mm^2^/S at pristine brick (CEs0%) to 0.2055 mm^2^/S with CEs7% composition owing to eggshell waste and the significant amount of amorphous phase formed. Likewise, the thermal effusivity drops from 812.185 to 565.0824 Ws¹´²/(m²K) as the percentage of eggshell grows from 0 to 10%, correspondingly, as the level of eggshell rises, as displayed in Fig. 7c. Concurrently, the tendency of specific heat capacity changed with the quantity of additive, peaking at 1.7790 MJ/m^3^K with CEs7% brick (See in Fig. 7d). A good insulator possesses a high specific heat capacity since it takes longer to absorb additional heat; beforehand, it heats up^95^. As previously stated, lower values of thermal diffusivity resulted in a drop in thermal conductivity and consequently a rise in the thermal insulation of clay bricks. The results reported here were consistent with the theory that materials with low thermal effusivity will retain heat for a longer period of time than materials with strong thermal resistance^96,97^. Thus, highly porous materials that are good thermal insulators have reduced thermal effusivity as their porosity grows^98^. The presence of eggshell wastes at the proper amount of 7%, which demonstrated remarkable properties, such as a huge surface area and high porosity throughout the improved clay composites, is the primary rationale for these results. Nevertheless, ion exchange occurs at lower eggshell concentrations (1–5%) when alkaline clay pore solutions of charged ions such as Ca^2+^, Mg^2+^, Na^+^, and OH^−^ form. Additionally, the unique surface area of the eggshell wastes effectively allows liquid to penetrate the interlayers^59^. However, increasing the proportion of eggshell wastes to 10% in clay production could not increase their effectiveness because too much mineral content increases CaCO_3_ clumping^99^. Aggregation caused eggshell minerals to lose their naturally occurring advantage of featuring a high specific surface area. As a result, they are no longer able to function as a location of nucleation, which speeds up the hydration and dispersion of porosity in clay mixtures and modifies the thermophysical properties of the clay substrate^100^. Since it retains its effectiveness inside the structure and enhances the performance of the clay compound, eggshell waste agglomeration with relatively modest levels and an enhanced specific surface area is chosen. Since each specimen’s thermal conductivity is less than 0.6 W/mK, all of them fulfil the necessary requirements^83^. Several prior investigations indicated the same pattern in improving the thermal performance of burnt clay bricks^101–103^.

In light of these observations, this significant drop, particularly in the CEs10% specimen, corresponds to a 50% decrease in thermal conductivity, a 30% reduction in thermal diffusivity, and a 30% decrease in thermal effusivity. Interestingly, the aforementioned apparent porosity, which likewise reached its peak at 10% eggshell content (i.e., 38.5%), was accompanied by the formation of micropores due to excessive organic component burning. This increase in porosity at higher eggshell levels corresponds directly to the observed decrease in thermal conductivity since micropores interrupt heat transfer channels, enhancing the brick’s thermal insulation qualities. Therefore, this relationship underscores the dual role of porosity in optimizing thermal properties: moderate porosity enhances material density and reduces heat conduction, while excessive porosity, as seen in the CEs10% specimen, promotes superior thermal insulation. This correlation provides a clearer understanding of the impact of eggshell content on the thermal performance of clay-based bricks.

Table 4 provides a comparative analysis of the bulk density, shrinkage, and thermal properties of clay-based materials reported in the literature and those obtained in this study. The inclusion of 10% eggshell waste as a filler within clay bricks optimized the bulk density to 1.56 g/cm^3^, shrinkage to 1.96%, and thermal conductivity to 0.3562 W/mK. Notably, the thermal conductivity achieved in this study is the lowest among the materials compared, a significant improvement attributed to the formation of micropores in the brick matrix during the sintering process. These micropores, created by the combustion of organic components in the eggshells, enhance the thermal insulation properties of the bricks. Furthermore, the firing temperature plays a critical role in improving thermal performance. Higher firing temperatures promote material densification and liquid phase formation, thereby reducing thermal conductivity. Additionally, the brick containing 10% eggshell waste, fired at 1100 °C, demonstrated a 50% reduction in thermal conductivity, highlighting its potential to lower the energy demands for heating and cooling in buildings^94^.

**Table 4:** A comparison of the bulk density, shrinkage, and other thermal features of clay-based materials to the current work.

No.	Clay based-material	Bulk density (g/cm^3^)	Shrinkage (%)	Thermal conductivity (W/mK)	Ref.
1	10% Eggshell waste	1.56	1.96	0.3562	Current work
2	20% Eggshell			0.65	^24^
3	1% Reduced graphene oxide (rGO)			0.33	^74^
4	rGO–Titanium dioxide (TiO_2_)			0.44	^83^
5	Date palm fiber			0.342	^104^
6	20% Granite Powder and 10% Eggshell Powder	1.76			^105^
7	20% Eggshell			0.36	^106^
8	20% Eggshell ash			1.23	^107^
9	10% Date palm waste			0.342	^108^
10	50% Eggshell	1.7	6		^109^
11	5% Bone Ash	1.59			^110^
12	7.5% Plastic	1.289	5		^111^
13	10% Expanded Perlite + 10% recycling paper sludge			0.432	^112^
14	3% Aluminum dross waste	1.307		0.32	^113^
15	Fly ash			0.40–0.45	^114^

## Conclusion

Fired clay bricks are crucial elements in construction, but they must meet specific requirements to be suitable for modern applications. These include being aesthetically appealing, robust, durable, and capable of offering low heat transfer rates. In this context, integrating eggshells as a CaCO₃ filler in fired clay bricks presents a promising strategy for reducing energy consumption and minimizing eggshell waste. This approach not only repurposes what would otherwise be a waste product but also contributes to reducing eggshell landfill pollution. The XRD analysis indicates that the addition of eggshells leads to the formation of siliceous melts, which enhance the thermal insulation properties of the bricks. The firing temperature plays a critical role in improving the mechanical and thermal properties of the final product. Specifically, the formation of wollastonite (CaSiO_3_) during the firing process contributes to the enhanced performance of the bricks. The EDX analysis provided important insights into the physicochemical changes resulting from the inclusion of eggshells, with the CEs7% brick (7% eggshell content) showing a 38.7% reduction in dry shrinkage, a 78.8% increase in average pore size, an apparent porosity of 52.7%, a bulk density of 1.66 g/cm³, and a compressive strength of 0.5 MPa compared to pristine bricks. In terms of thermal performance, the brick containing 10% eggshells and fired at 1100^°^C exhibited the most significant reductions in heat conductivity (i.e., 50%), thermal diffusivity (i.e., 30%), and thermal effusivity (i.e., 30%) compared to the pristine brick. This variation demonstrates the way the mineral content of clay has an important effect on brick qualities, regardless of whether the same waste products and proportions are used. Despite these promising findings, it is important to note some limitations, including the relatively low compressive strength (0.5 MPa) of the eggshell-modified bricks. Further ongoing tests, such as long-term durability studies and the assessment of other mechanical properties, are essential to fully understanding the performance of these modified bricks in real-world construction environments. In summary, bricks composed of a clay and eggshell mixture show great potential as thermal insulators, offering a sustainable solution for building materials. Future research should focus on optimizing the eggshell content and firing conditions to improve the mechanical strength while maintaining the excellent thermal insulation properties observed in this study.

## Data Availability

All data generated or analysed during this study are included in this published article.
